# Performance analysis of mouldboard plough body with raised elements and frame based on numerical simulation

**DOI:** 10.1371/journal.pone.0331839

**Published:** 2025-09-11

**Authors:** Xueting Ma, Xinli Wang, Chang Wan, Ganggang Guo, Jinfei Zhao, Quanzhong Zhang

**Affiliations:** 1 College of Mechanical and Electrical Engineering, Tarim University, Alar, China; 2 Modern Agricultural Engineering Key Laboratory at Universities of Education Department of Xinjiang Uygur Autonomous Region, Tarim University, Alar, China; 3 Xinjiang Production and Construction Corps (XPCC) Key Laboratory of Utilization and Equipment of Special Agricultural and Forestry Products in Southern Xinjiang, Tarim University, Alar, China; China Construction Fourth Engineering Division Corp. Ltd, CHINA

## Abstract

This paper designed a mouldboard plough device, and performs performance analysis on the share-type plow body and frame based on numerical simulation methods. Firstly, the effects of forward speed, lugs angle, and the radius of the raised structure on the mouldboard plough’s performance were investigated, utilizing the discrete element method. The effects of these variables are analyzed through the response surface method. Furthermore, the significance of each factor is examined, and optimal parameter combinations are identified. The degree of influence on soil penetration resistance, ranked from largest to smallest, is as follows: forward speed, lugs angle, and mouldboard plough surface convex radius. Specifically, resistance to soil penetration increases with an increase in forward speed, lugs angle, and the radius of the convex structure. The degree of influence on the number of soil disturbance particles is ranked as follows: forward speed, the radius of the convex structure, and lugs angle. The number of soil disturbance particles decreases with an increase in forward speed, increases with a larger radius of the convex structure, and slightly decreases with an increase in the lugs angle. By establishing a regression model, the optimal parameter combination for the mouldboard plough was determined to be a forward speed of 0.8 m/s, a lugs angle of 45°, and a convex structure radius of 9 mm. Then, based on finite element analysis, both static and modal analyses were conducted on the frame of the plow device. The results indicated that, during stable operation of the mouldboard plough, the maximum stress occurs at the hinge joint between the frame and the tractor. The maximum stress value recorded is 24.7 MPa, with a corresponding maximum deformation of 0.02 mm, demonstrating that the designed frame satisfies the static requirements. Furthermore, the first-order natural frequency of the frame is 92 Hz, which is significantly higher than the external excitation frequency, thereby preventing the occurrence of resonance. Finally, the harmonic response analysis was performed on the frame, and the results showed that under the excitation conditions of 90 Hz and 500 Hz, the maximum displacement of the rack was 16.5 mm, and the results showed that it would not affect the working performance of the machine.

## 1. Introduction

Land cultivation is a fundamental operation in agricultural planting. This process involves turning and loosening the soil to create suitable conditions for sowing. Among the various tools available, the mouldboard plough is noted for its superior soil-turning performance and is widely utilized. The mouldboard plough effectively turns the surface soil, incorporating weeds, stubble, and fertilizers into the furrow’s bottom while bringing deeper soil to the surface layer. To enhance the mouldboard plough’s performance, numerous scholars have conducted extensive research, primarily focusing on optimizing the structural and working parameters of the mouldboard plough body to minimize farming resistance, reduce soil adhesion, and decrease wear and tear [1–3]. Sun J.F et al. [4] integrated a bear claw structure into the mouldboard plough body, resulting in the design of a new type of plowing device. They examined the influence of factors such as angle, rotation speed, forward speed, and plowing depth, while using power and energy consumption as response indicators to explore its optimal performance and identify the best parameter combination. Liao QX et al. [5] proposed a land preparation method that involves active plowing followed by double-edged rotary tillage. Utilizing the sliding shear principle, they determined the key structural parameters for a double-edged rotary tiller featuring both long and short blades.

To achieve the optimization of the structural and working parameters of the mouldboard plough, the discrete element method is frequently employed in mouldboard plough studies. This method has gained prominence in recent years for simulating the interactions between soil and agricultural machinery, effectively illustrating the movement trajectories of soil particles and their contact dynamics [6,7]. Zheng X et al. [8] utilized EDEM and RecurDyn to investigate the influence of various structural forms on mouldboard plough-soil adhesion. The study identified the optimal parameter combination and validated the correlation between these parameters through both field tests and simulation comparisons. Shi YL et al. [9] designed a plowshare furrowing device for a sweet potato ridging and shaping machine. They utilized EDEM to develop an interaction model between the plowshare furrowing device and the soil, employing the discrete element method. This process allowed them to determine the optimal combination of installation angle, dozing angle, and unit line angle difference.

This paper designed a mouldboard plough device, and performs performance analysis on the share-type plow body and frame based on numerical simulation methods. Based on our observations in daily life, certain insect wings exhibit protrusions on their surfaces that can effectively reduce drag. Consequently, we propose the integration of these raised elements into the design of moldboard plows. The study analyzes how the forward speed of the mouldboard plough, the soil penetration angle, and the bulge radius of the mouldboard plough body influence soil disturbance and working resistance. Based on the research conducted above, the performance of the frame was analyzed using the finite element method to assess whether the maximum stress experienced by the frame during operation exceeds the allowable limits and to evaluate the potential for resonance. The findings of this research may serve as a valuable reference for the design of mouldboard plough device.

## 2. Materials and methods

### 2.1. Materials

**Fig 1:**
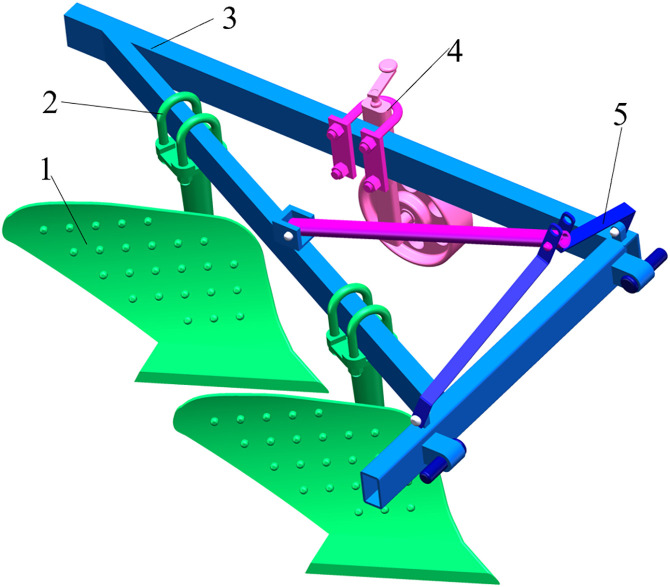
The overall structure of the device. 1 is mouldboard plough; 2 is U-shaped bolts; 3 is frame; 4 is depth limiting wheel; 5 is three-point suspension.

The structure of the mouldboard plough device examined in this article is illustrated in Fig 1. The device primarily comprises a plow, U-bolts, a frame, a depth-limiting wheel, a three-point suspension, and other components. Notably, the frame is connected to the tractor via a three-point suspension, allowing the tractor’s traction to pull the entire machine forward. The depth of soil penetration can be adjusted by modifying the position of the depth-limiting wheel.

**Fig 2:**
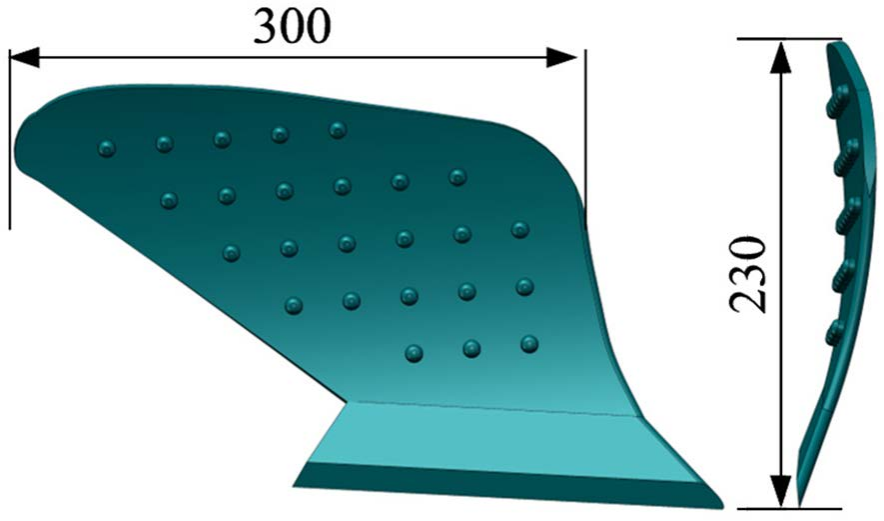
The composition of the body of a mouldboard plough. 1 is flow share; 2 is moldboard; 3 is tibial blade line; 4 is top edge line; 5 is wing edge line; 6 is wing line; 7 is share edge line.

The mouldboard plough body serves as the primary soil-contacting component during the plowing process, and its performance directly influences both the effectiveness of plowing and energy consumption. The primary methods for shaping the surface of the mouldboard plough body include the curved element line method, the horizontal straight element line method, and the inclined element line method.The design of the mouldboard plough is illustrated in Fig 2. The mouldboard plough body surface is primarily composed of two parts: the plow share and the moldboard. The bottom edge line of the mouldboard plough body’s curved surface is referred to as the share edge line. The right edge line is known as the tibial edge line, the top edge line is simply called the top edge line, and the left edge line consists of two components: the wing edge line and the wing line. This study focuses on the body model of a mouldboard plough that incorporates raised elements as the primary research subject. This raised structure effectively enhances the soil crushing rate of the mouldboard plough when operating in wet soil conditions. The working width of the mouldboard plough is approximately 300 mm, while the plowing depth is around 220 mm. The surface of the moldboard incorporates 25 evenly spaced raised elements, each of which is hemispherical in shape.

### 2.2. Methods

This study presents a simulation analysis of a mouldboard plough body utilizing the discrete element method based on EDEM 2025 (Altair Engineering, Inc., Troy, USA) [10]. Initially, the displacement of soil particles during the operation of the plow was analyzed, and the displacement characteristics of soil particles at different positions were explored. Then, a single-factor test was performed to investigate the effects of three variables—the forward speed of the mouldboard plough, the angle of entry of the mouldboard plough into the soil and the radius of the raised structure of the mouldboard plough—on the working resistance of the mouldboard plough body and the extent of soil disturbance. Subsequently, employing the response surface method, a regression equation was derived to describe the relationship between working resistance and soil disturbance. The significance of the three factors was examined, leading to the identification of the optimal parameter combination [11,12]. Based on the simulation results of the mouldboard plough, static and modal analyses were conducted on the frame using ABAQUS 6.14 (Dassault Systemes, Vélizy-Villacoublay, France) to assess whether the maximum stress experienced by the frame exceeded the yield stress of the material, as well as to evaluate the potential for resonance during operation.

**Fig 3:**
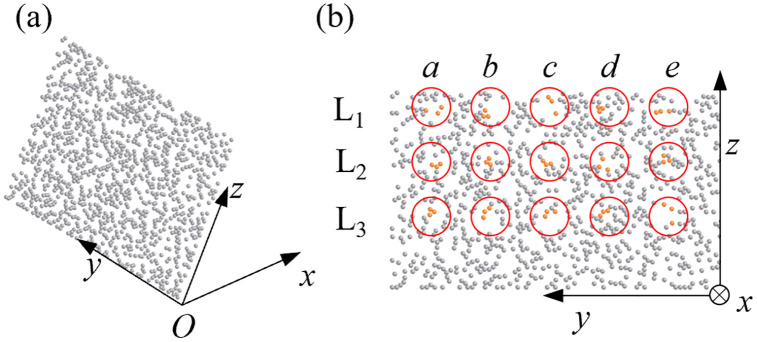
Selection of soil particle sampling points. (a) Selection of profile; (b) Selection of sampling points.

The soil particles undergo a certain degree of displacement before, during, and after the treatment with the mouldboard plough. To analyze the displacement of soil particles during the mouldboard plough operation, the Clipping function in the EDEM post-processing module was utilized to create a plane perpendicular to the forward direction, as illustrated in Fig 3. This plane was approximately located in the middle of the soil bin and was divided into three layers: upper, middle, and lower. Five sampling points were selected within each layer using the Manual Selection function in the Setup Selections of EDEM. Specifically, the particles in column c were situated in the center of the mouldboard plough operation area, while those in columns a and e were located at the edges. The particles in columns b and d were positioned approximately at the midpoints between columns a and c, and columns c and e, respectively. At each sampling point, three particles were selected, and their displacements in the x, y, and z directions were tracked and analyzed.

This study considers three influencing factors: the forward speed of the mouldboard plough, the lugs angle, and the bulge radius of the mouldboard plough body. The responses measured are the working resistance of the mouldboard plough and the degree of soil disturbance. A single-factor experimental design was developed based on these parameters. Following actual operational requirements, the soil penetration depth of the mouldboard plough is established at 220 mm. The rationale behind the single-factor experimental design is as follows:

(1) Maintain a constant lugs angle and the radius of the convex structure while varying the forward speed of the mouldboard plough at 0.4 m/s, 0.8 m/s, and 1.2 m/s. The objective is to investigate the effects of different forward speeds on the working resistance of the mouldboard plough, as well as the influence on the number of soil particles disturbed.
(2) Maintaining a constant radius for the convex structure and a consistent forward speed, the lugs angles of the mouldboard plough were set to 45°, 48°, and 51°, respectively. The objective is to investigate the effects of varying penetration angles on both the working resistance of the mouldboard plough and the quantity of soil particles disturbed.
(3) Maintain the forward speed and soil penetration angle constant while adjusting the convex structure radii of the mouldboard plough to 9 mm, 15 mm, and 21 mm, respectively. Investigate the effects of varying convex structure radii on both the working resistance of the mouldboard plough and the quantity of disturbed soil particles.

To further investigate the optimal parameter combination of the three influencing factors, a multi-factor orthogonal rotation combination test was designed. The factor level coding is presented in Table 1. Using the working resistance of the mouldboard plough and the number of soil disturbance particles as responses, a regression model was developed. This model, in conjunction with the constraint equations, was utilized to determine the optimal parameter combination.

**Table 1:** Test factor coding.

Code	Forward speed A/m·s-1	lugs angle B/°	Raised structure radius C/mm
1	0.4	45°	9 mm
0	0.8	48°	15 mm
−1	1.2	51°	21 mm

Static analysis refers to the force analysis of a structure subjected to a constant static load, without accounting for the effects of inertia and damping. This method is primarily employed to evaluate whether the maximum stress and displacement of the object under investigation remain within acceptable limits during steady-state operation. Specifically, the maximum stress on the frame should not surpass the allowable stress, the calculation of which is detailed in Equation (1). Utilizing the discrete element method to determine the working resistance of the mouldboard plough, a static analysis of the frame is conducted. Should the maximum stress or maximum displacement exceed the acceptable thresholds, optimization of the frame is necessary.


$$[\sigma ]=\frac{\sigma_{s}}{n_{s}}$$ (1)

In the formula, [σ] represents the allowable stress of the material in megapascals (MPa), while σ_s_ denotes the yield limit of the material (MPa). The safety factor for the complete machine is denoted as n_s_, which is set to 2. Consequently, the allowable stress is calculated to be 117.5 MPa.

In the design of the frame, it is essential to determine the natural frequency and vibration mode to prevent resonance issues. Modal analysis serves as an effective method for identifying both the natural frequency and vibration shape. Therefore, during the design stage, modal analysis can provide guidance for solving resonance problems. When external excitation approaches the natural frequency of the frame, resonance may occur. The mathematical model for the finite element modal analysis is presented in Equation (2).


$$\left[M\mathrm{\backslash rightleft}\{\ddot{X}\right\}+\left[C\right]\left\{\dot{X}\right\}+\left[K\right]\left\{X\right\}=\left\{F\left(T\right)\right\}$$ (2)

In the formula, [M] is the mass matrix, [C] is the damping matrix, and {X} is the displacement matrix.

## 3. Discrete element model construction

### 3.1. Construction of soil particle model

**Fig 4:**
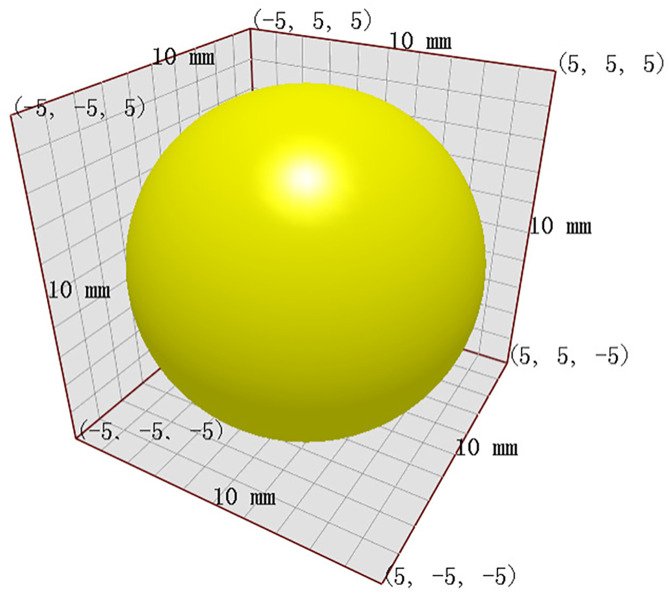
Soil particle simulation model.

In EDEM software, the primary parameters required for simulation experiments include particle parameters, material parameters, and contact parameters. Based on relevant literature [13], the soil particle radius is established at 5.00 mm. The rolling friction factor and static friction factor between particles are set at 0.15 and 0.8, respectively. The density is defined as 1680 kg/m³, the recovery coefficient is set to 0.55, the soil shear modulus is established at 1 × 10^6 Pa, and Poisson’s ratio is set to 0.41 (Fig 4).

### 3.2. Contact model determination

**Fig 5:**
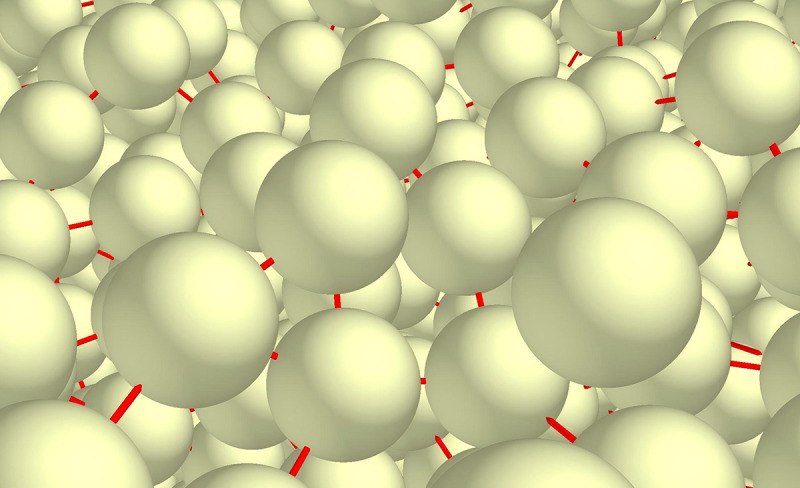
Bonding bonds between soil particles.

In this study, the contact model between the soil and mouldboard plough body was determined to be the Hertz-Mindlin (no slip) model, while the soil-soil contact model was selected as the Hertz-Mindlin with Bonding model (Fig 5). The Hertz-Mindlin with Bonding model is frequently employed to simulate bonded particles. In this model, the particles are interconnected through Bond bonds, which can withstand specific forces and moments. When the applied force or torque exceeds a certain threshold, the bonding bonds break, resulting in the separation of the bonded particles. Once the particles are separated, the Bond bonds are not reformed. This model effectively represents the soil-breaking process [14–16].

There are five primary bonding parameters: normal stiffness, tangential stiffness, critical normal stress, critical tangential stress, and bonding radius. Among these, critical stress serves as a crucial indicator for determining whether a bond is broken. Its value is closely associated with bond strength and directly influences the extent of soil fragmentation and operational resistance in simulations. Based on relevant literature [13], the bond normal stiffness is established at 1 × 10^8 N/m³, the tangential stiffness at 5 × 10^7 N/m³, the critical normal stress at 18.45 kPa, and the critical tangential stress at 18.58 kPa. The bonding radius is set to 5.45 mm.

Following the assessment of the aforementioned parameters, the soil trench model can be constructed. In conjunction with the dimensions of the mouldboard plough, soil bin designed in this study measures 1000 mm × 1500 mm × 400 mm.

### 3.3. Construction of the mouldboard plough model

**Fig 6:**
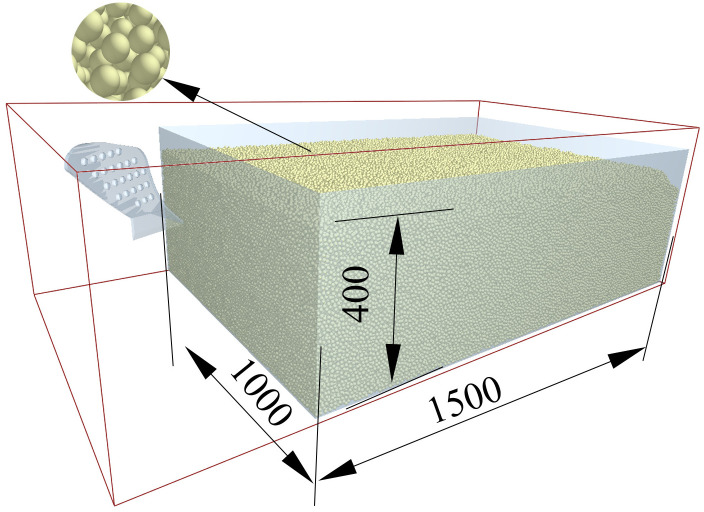
Simulation model.

This study utilizes SolidWorks software to create a three-dimensional simulation model of the mouldboard plough body. The model is then converted into STL format and imported into EDEM software for further analysis. After importing the model, adjust the position of the mouldboard plough within the soil trench. Ensure that the mouldboard plough is situated on the left side of the trench, maintaining a proximity to it without making contact. This positioning will allow the mouldboard plough to subsequently move to the right at a specified speed. The material selected for the mouldboard plough model is 65Mn, which is commonly used for mouldboard plough bodies, and the depth of penetration into the soil is set at 22 cm. The material has a density ρ of 7810 kg/m^3^, a shear modulus of 1 × 10^10^ Pa, and a Poisson’s ratio of 0.3. Fig 6 shows the constructed simulation model.

Table 2 is a summary of the above simulation parameters.

**Table 2:** Simulation parameters [13].

Parameter	Numerical value	Parameter	Value
Soil tank size/mm	1000 × 300 × 400	Soil-soil rolling friction coefficient	0.15
Buried depth/mm	220	Soil-soil static friction coefficient	0.8
soil particle density *ρ*_1_(kg·m^-3^)	1680	Soil-65Mn collision recovery coefficient	0.45
Poisson’s ratio of soil particles *v*_1_	0.41	Soil-65Mn static friction coefficient	0.31
Soil particle shear modulus *G*_1_/Pa	1 × 106	Soil-65Mn rolling friction coefficient	0.125
Soil particle radius *r*/mm	5	Soil normal stiffness/(N·m^-3^)	1 × 108
65Mn density *ρ*_2_/(kg·m^-3^)	7861	Soil tangential stiffness/(N·m^-3^)	5 × 107
Poisson’s ratio of 65Mn	0.3	Critical normal stress/kPa	18.45
65Mn shear modulus/Pa	7.9 × 1010	Critical tangential stress/kPa	18.58
Soil-soil collision recovery coefficient	0.55	Bonding radius/mm	5.45

## 4. Analysis of the movement of soil particles

**Fig 7:**
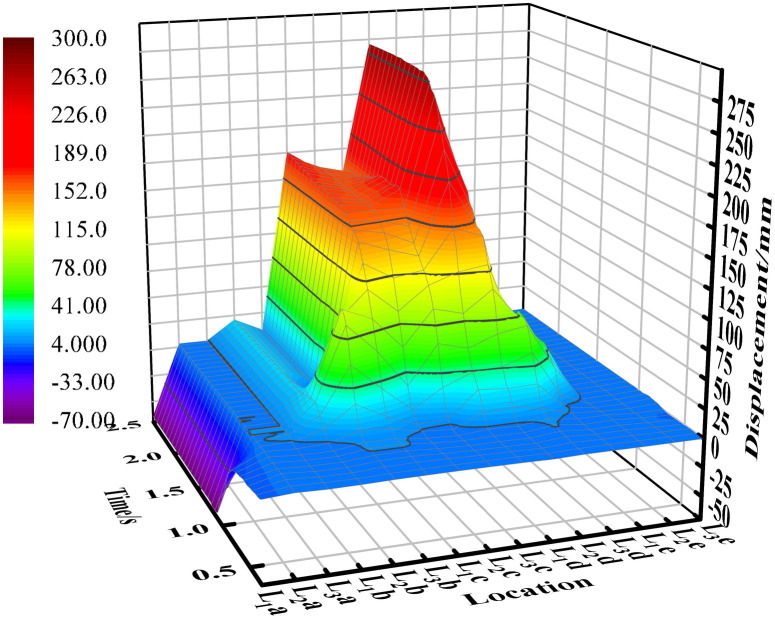
Displacement of soil particles in x direction.

Fig 7 illustrates the x-direction displacement of soil particles at each sampling point. The figure indicates that, overall, the displacement of most particles in the x-direction is positive, with only the particles at the two edge sampling points (L_1_a and L_2_a) exhibiting negative displacement. This phenomenon can be attributed to the bonding forces between soil particles, which form a cohesive mass. As the mouldboard plough advances, it generates torque on the soil, causing some edge particles to move backward. Additionally, it was observed that the displacement of soil particles is most pronounced in the centerline area of the mouldboard plough ‘s working zone. This is because the particles in this area are pushed by the mouldboard plough for the longest time during the mouldboard plough operation, while those near the edges tend to exit the mouldboard plough ‘s influence earlier, resulting in reduced forward movement.

**Fig 8:**
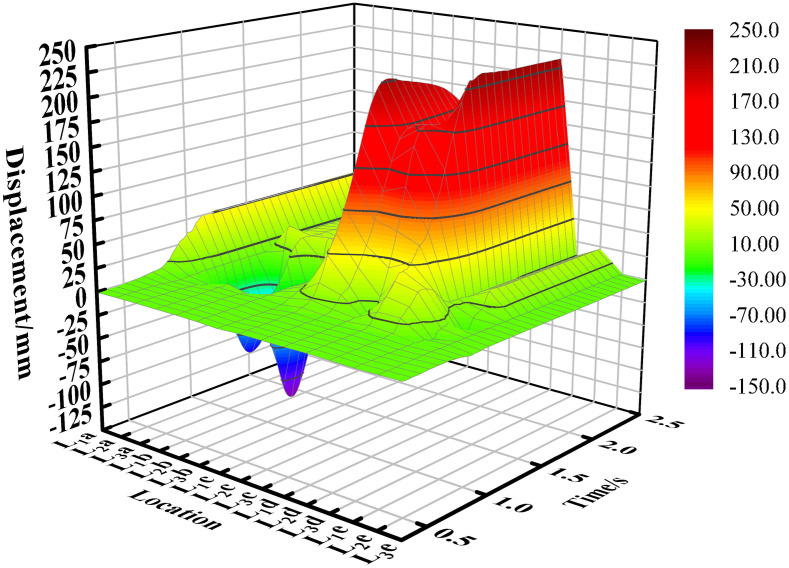
Displacement of soil particles in y direction.

Fig 8 illustrates the y-direction displacement of soil particles at each sampling point. The figure indicates that the displacement of soil particles located on one side of the concave surface of the mouldboard plough is in the positive direction. Soil particles on the convex side of the mouldboard plough move first in the negative direction and then in the positive direction. This behavior can be attributed to the force exerted by the former on the concave curved surface of the mouldboard plough, which acts in the positive direction of the y-axis, while the latter moves in the negative direction of the y-axis under the action of the convex surface of the mouldboard plough. Furthermore, the area disturbed by the plow creates a groove, with the slopes on either side exhibiting inconsistency. The slope adjacent to the sampling point in column A is steeper, which causes particles that initially moved in the negative direction to shift towards the positive direction along the steep slope for a certain distance, influenced by their own gravitational force after reaching this area.

**Fig 9:**
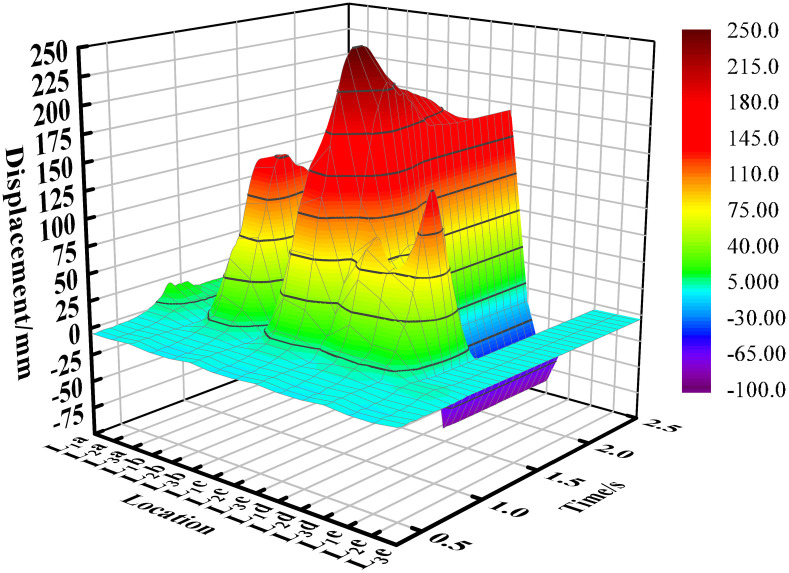
Displacement of soil particles in z direction.

Fig 9 illustrates the z-direction displacement of soil particles at each sampling point. The figure indicates that, overall, the displacement directions of the particles are inconsistent. While some displacements are positive, others are negative, the majority of soil particles exhibit a positive displacement. Notably, the displacement of soil particles is more pronounced near the center of the mouldboard plough’s working area, whereas it is less pronounced near the edges. Some particles initially display a positive displacement before transitioning to a negative displacement (as observed in the soil particles in column d). This behavior can be attributed to the upward force exerted by the curved surface of the mouldboard plough, which initially lifts the particles, followed by their subsequent rolling back into the soil layer under the influence of gravity and other soil disturbances. Additionally, during plowing operations, the bonding forces among soil particles are disrupted, resulting in increased looseness of the soil, which contributes to an overall rise in the height of the soil particles.

## 5. Single-factor test results and analysis

### 5.1. Effect of forward speed on working resistance

Three traveling speeds of 0.5 m/s, 1.0 m/s, and 1.5 m/s were established for the mouldboard plough body. The resistance encountered by the mouldboard plough body at these varying speeds were compared.

**Fig 10:**
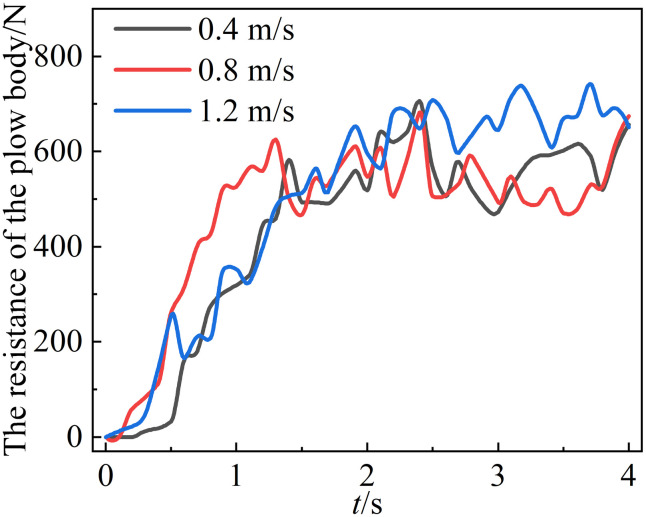
Changes in mouldboard plough body resistance at different forward speeds.

Fig 10 illustrates that the soil resistance experienced by the mouldboard plough body varies with different forward speeds during simulation experiments. Overall, the resistance initially rises rapidly as the mouldboard plough body enters the soil, followed by a gradual decrease in growth rate, ultimately stabilizing within a certain range. This behavior can be attributed to the stable and continuous process of the mouldboard plough body cutting into the soil, where the topsoil is consistently squeezed and displaced, resulting in a regular periodic state. Consequently, the mouldboard plough body encounters a certain level of fluctuations in resistance. Furthermore, Fig 6 indicates that the resistance of the mouldboard plough body increases with speed. At a speed of 1.2 m/s, the mouldboard plough body experiences significant resistance, with an average stabilized resistance of 703.32 N. Conversely, at a speed of 0.4 m/s, the resistance is comparatively lower, averaging 612.51 N after stabilization.

### 5.2. Effect of lugs angle on mouldboard plough body resistance

Three lugs angles of 51°, 48°, and 45° were established for the mouldboard plough body, and the resistance encountered by the mouldboard plough body, were compared under these varying penetration angle conditions.

**Fig 11:**
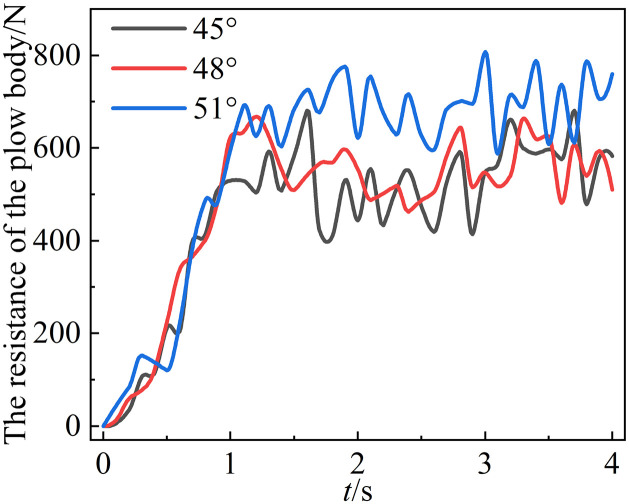
Changes in mouldboard plough body resistance at different lugs angles.

Fig 11 illustrates that the mouldboard plough body experiences greater resistance when operating at a lugs angle of 51°, with an average resistance of 684.23 N. In contrast, when the mouldboard plough body operates at a lugs angle of 45°, the resistance is reduced, yielding an average resistance of 597.31 N.

### 5.3. Effect of lugs angle on mouldboard plough body resistance

**Fig 12:**
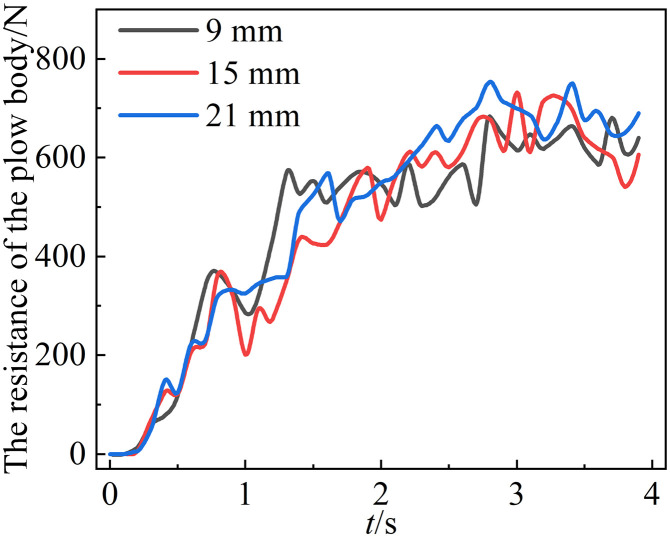
Mouldboard plough body resistance diagram with different mouldboard plough surface convex structure radii.

Fig 12 illustrates that the average working resistance of the mouldboard plough body with a raised structure radius of 21 mm is greater, measuring 687.20 N, compared to the average working resistance of the mouldboard plough body with a raised structure of 9 mm, which is 604.45 N. This indicates a positive correlation between the radius of the convex structure and the working resistance of the mouldboard plough body.

## 6. Multi-factor simulation test results and analysis

The orthogonal rotation experimental method was employed to conduct simulation experiments, with various influencing factors designated as independent variables. Soil resistance and the number of disturbed soil particles encountered by the mouldboard plough during the simulation were utilized as evaluation standards. The test results are presented in Table 3. This paper counts the number of particles with a soil particle velocity greater than 0.24 m/s to characterize the degree of soil disturbance.

**Table 3:** Multi-factor experimental results.

No.	A/m·s^-1^	B/°	C/mm	f/N	N
1	0.8	48	15	824.7	569191
2	0.8	45	9	815.4	568612
3	0.8	48	15	826.5	568519
4	0.8	48	15	825.76	569226
5	1.2	45	15	857.65	561890
6	0.8	51	21	833.23	569214
7	0.4	48	21	782.44	580779
8	0.4	51	15	786.33	579918
9	0.8	51	9	827.74	568591
10	0.4	48	9	776.44	578574
11	0.8	45	21	829.22	569227
12	0.4	45	15	773.83	579936
13	1.2	51	15	867.29	561847
14	1.2	48	9	860.13	560072
15	0.8	48	15	824.37	568579
16	1.2	48	21	867.33	562883
17	0.8	48	15	826.32	569191

Note: A represents the forward speed of the mouldboard plough, B represents the lugs angle, and C represents the bulge radius of the mouldboard plough body, the same as below.

The quadratic polynomial regression model of the working resistance of the mouldboard plough and the number of soil disturbance particles, as presented in Equations (3) and (4), was derived using Design–Expert 10.0 software.


$$\text{Y1}=\text{607}.\text{28 }+\text{ 176}.\text{24A }+\text{ 0}.\text{84B }+\text{ 5}.\text{64C }-\text{ 0}.\text{60AB }+\text{ 0}.\text{12AC }-\text{ 0}.\text{12BC }-\text{ 28}.\text{34}{\text{A}}^{\text{2}}\;+\text{ 0}.\text{03}{\text{B}}^{\text{2}}\;+\text{ 0}.\text{02}{\text{C}}^{\text{2}}$$ (3)

$$\text{Y2}=\text{636760}.\text{13}-\text{41753}.\text{56A}-\text{1852}.\text{93B}+\text{197}.\text{10C}-\text{5}.\text{21AB}+\text{63}.\text{13AC}+\text{0}.\text{11BC}+\text{11497}.\text{34}{\text{A}}^{\text{2}}+\text{19}.\text{29}{\text{B}}^{2}-\text{4}.\text{09}{\text{C}}^{2}$$ (4)

In the formula, Y1 represents the resistance of the mouldboard plough, Y2 denotes the number of soil disturbance particles, A indicates the forward speed, B signifies the soil penetration angle, and C refers to the radius of the convex structure.

The results of the analysis of variance concerning work resistance are presented in Table 4. The analysis reveals that the p-value for the regression model is less than 0.05, indicating the model’s significance. Additionally, the p-value for the lack-of-fit term exceeds 0.05, suggesting a good fit for the experimental data. Notably, experimental factors A, B, C, BC, and A^2^ significantly influence work resistance, whereas the remaining regression items do not exhibit a significant impact.

**Table 4:** ANOVA for the quadratic polynomial regression model of mouldboard plough body working resistance.

Source	Sum of squares	Degrees of freedom	Mean square	F-value	p-value
Model	14315.24	9	1590.58	777.82	< 0.0001**
A	13891.11	1	13891.11	6792.99	< 0.0001**
B	185.19	1	185.19	90.56	< 0.0001**
C	132.11	1	132.11	64.61	< 0.0001**
AB	2.04	1	2.04	1.0000	0.3506
AC	0.3600	1	0.3600	0.1760	0.6874
BC	17.35	1	17.35	8.48	0.0226**
A^2^	86.55	1	86.55	42.32	0.0003**
B^2^	0.3272	1	0.3272	0.1600	0.7011
C^2^	1.46	1	1.46	0.7137	0.4261
Residual	14.31	7	2.04		
Lack of fit term	10.66	3	3.55	3.89	0.1111
Pure error	3.65	4	0.9131		
Cor Total	14329.55	16			

Note: * indicates a significant difference (*p* < 0.05); ** indicates an extremely significant difference (*p* < 0.01), the same as below.

The results of the analysis of variance concerning the number of soil-disturbed particles are presented in Table 5. The analysis reveals that the p-value for the regression model is less than 0.05, indicating the model’s significance. Additionally, the p-value for the lack-of-fit term exceeds 0.05, suggesting a good fit for the experimental data. Notably, experimental factors A, C and A2 significantly influence work resistance, whereas the remaining regression items do not exhibit a significant impact.

**Table 5:** ANOVA for the quadratic polynomial regression model of several soil-disturbed particles.

Source	Sum of squares	Degrees of freedom	Mean square	F-value	p-value
Model	6.768 × 10^8^	9	7.521 × 10^7^	235.84	< 0.0001**
A	6.573 × 10^8^	1	6.573 × 10^8^	2061.29	< 0.0001**
B	1128.13	1	1128.13	0.0035	0.9542
C	4.889 × 10^6^	1	4.889 × 10^6^	15.33	0.0058**
AB	156.25	1	156.25	0.0005	0.9830
AC	91809.00	1	91809.00	0.2879	0.6082
BC	16.00	1	16.00	0.0001	0.9945
A^2^	1.425 × 10^7^	1	1.425 × 10^7^	44.68	0.0003**
B^2^	1.269 × 10^5^	1	1.269 × 10^5^	0.3978	0.5482
C^2^	91202.02	1	91202.02	0.2860	0.6093
Residual	2.232 × 10^6^	7	3.189 × 10^5^		
Lack of fit term	1.794 × 10^6^	3	5.980 × 10^5^	5.46	0.0673
Pure error	4.380 × 10^5^	4	1.095 × 10^5^		
Cor Total	6.791 × 10^8^	16			

Based on the Design–Expert10.0 software, the response surface diagram of the mouldboard plough body working resistance and the number of soil disturbance particles is obtained to analyze the interaction between influencing factors [12,17,18].

**Fig 13:**
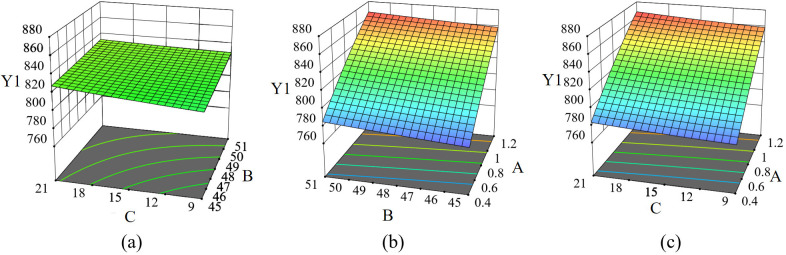
Mouldboard plough body working resistance response surface. A is the forward speed, m/s; B is the lugs angle, °; C is the radius of the convex structure, mm; Y1 is the working resistance, N.

The response surface diagram illustrating the working resistance of the mouldboard plough body (Fig 13) indicates a positive correlation between working resistance and three factors: forward speed, lugs angle, and the radius of the convex structure. Notably, the influence of forward speed on working resistance is particularly pronounced.

**Fig 14:**
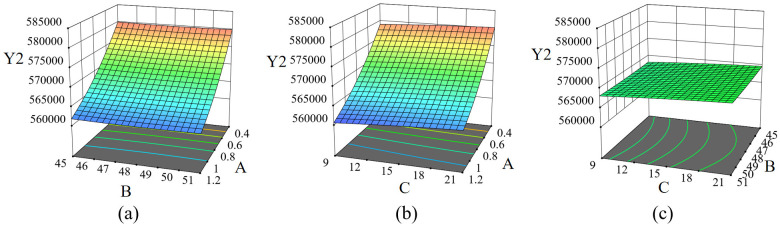
Soil disturbance response surface curved surface. A is the forward speed, m/s; B is the lugs angle, °; C is the radius of the convex structure, mm; Y1 is the working resistance, N.

The soil disturbance response surface diagram (Fig 14) illustrates that the number of disturbed soil particles is negatively correlated with forward speed, while a positive correlation exists between the radius of the convex structure and the lugs angle. Notably, the effect of forward speed on the number of disturbed soil particles is more pronounced, whereas the influence of the lugs angle on the number of disturbed particles is comparatively weaker.

Through the obtained regression model, the parameters of the opener are optimized to achieve excellent operating results. Its constraints and objective functions are shown in Equation (5).


$$\{\begin{array}{c} {}*20c\min f\left(A,B,C\right)\\ \max N\left(A,B,C\right)\\ 0.4\leq A\leq 1.8\\ 45\leq B\leq 51\\ 9\leq C\leq 21 \end{array}$$ (5)

Through optimization, the optimal parameter combination was determined: a forward speed of 0.4 m/s, a lugs angle of 45°, and a raised structure diameter of 15 mm. Under these conditions, the model predicts a working resistance of 773.83 N and a total of 579,941 soil disturbances.

## 7. Static and modal analysis of the frame

**Fig 15:**
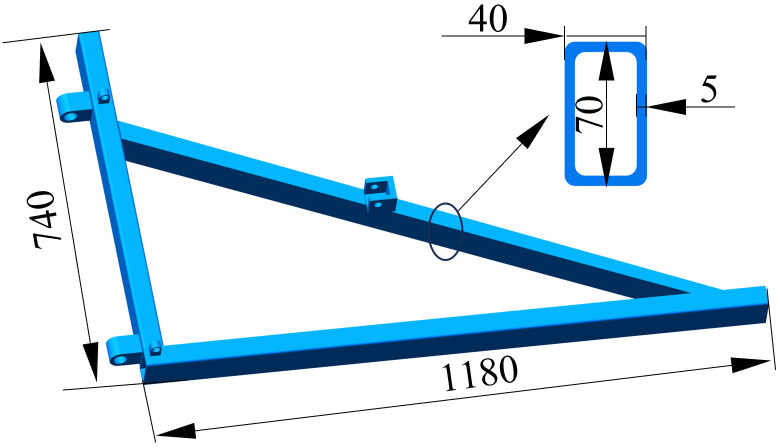
Frame structure and key dimensions.

The frame’s structure and key dimensions utilized for static and modal analysis are illustrated in Fig 15. Throughout its operation, the frame is primarily constrained by the three-point suspension system. The forces acting on the frame consist mainly of its own weight, the working resistance of the mouldboard plough, and the weight of the mouldboard plough itself. The entire frame is composed of rectangular tubes made from Q235 steel. This material is known for its excellent plasticity, toughness, and welding characteristics. The elastic modulus (E) of Q235 is 206 × 10^3 MPa, the density (ρ) is 7850 kg/m³, the Poisson’s ratio (μ) is 0.3, and the yield strength is 235 MPa.

### 7.1. Static analysis results and analysis

**Fig 16:**
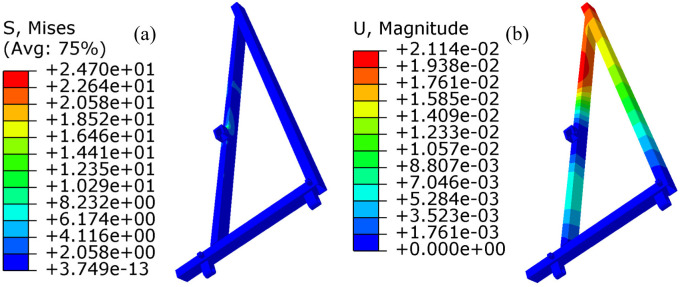
Static analysis results. (a) is the stress cloud diagram; (b) is the displacement cloud diagram.

The static analysis results of the frame are presented in Fig 16. These results indicate that, during stable operation, the maximum stress in the frame occurs at the midpoint where the two mouldboard ploughs are connected, at the articulation point with the tractor. The stress value recorded is 24.7 MPa, which is below the allowable stress. Additionally, the maximum displacement resulting from the force deformation of the frame is measured at 0.02 mm, a value that is negligible and will not adversely affect the frame’s performance. This evidence demonstrates that the designed frame satisfies the static requirements.

### 7.2. Modal analysis results and analysis

By analyzing the excitation characteristics of each vibration source associated with the frame, we can determine whether the natural frequency of the frame falls within the range of external excitations, which will inform the necessity of further optimizing the rack’s design. During operation, the device is primarily stimulated by the ground and the tractor.

The magnitude of the excitation generated by agricultural machines is largely influenced by the unevenness of the ground and the speed at which the machines traverse it. The excitation calculation is presented in Equation (6). Based on our calculations, the field excitation frequency ranges from 4 to 6 Hz.


$$f_{0}=\frac{1000v_{m}}{\lambda }$$ (6)

In the formula, *f*_0_ is the ground excitation frequency, Hz; *v*_*m*_ is the walking speed, m/s; *λ* is the wavelength of the uneven ground, generally 320 mm.

When the tractor is required to drive additional rotating devices in conjunction with the mouldboard plough, the high-speed rotating power output shaft induces a specific excitation effect on the frame. The calculation formula for the excitation frequency, denoted as f1, is presented in Equation (7).


$$f_{1}=\frac{n}{60}$$ (7)

In the formula, *f*_1_ is the tractor excitation frequency, Hz; *n* is the tractor power output shaft speed, r/min, here it is 1000 r/min.

**Fig 17:**
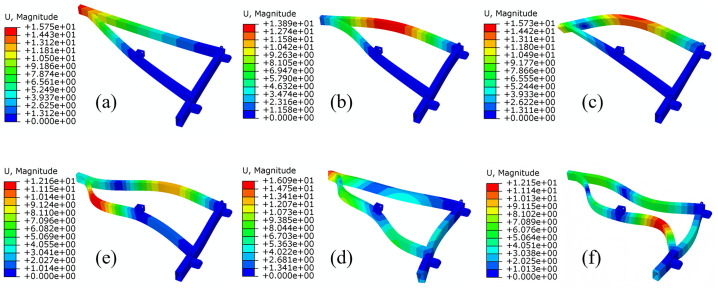
Mode shapes of the first six modes. (a) is the first-order mode; (b) is the second-order mode; (c) is the third-order mode; (d) is the fourth-order mode; (e) is the fifth-order mode; (f) is the sixth-order mode. order mode.

To determine the vibration characteristics of the frame, modal analysis was conducted on its components. Given that lower-order modes significantly influence the dynamic properties of the structure, the first six natural frequencies and their corresponding mode shapes were identified through this analysis. If the results for these first six orders meet the established criteria, no modifications will be necessary. The outcomes of the modal analysis are presented in Fig 17 and Table 6. Notably, among the first six natural frequencies, the lowest value recorded is 92 Hz, which is considerably higher than the excitation frequencies imposed on the frame. Specifically, the excitation frequency generated by the road spectrum ranges from approximately 4–6 Hz, while that produced by the tractor’s PTO shaft is about 17 Hz. Consequently, the likelihood of resonance occurring is minimal.

**Table 6:** Characteristics of the first six vibration modes of the frame.

Mode	Frequency/Hz	Vibration characteristics	Maximum deformation position	Maximum displacement/mm
1	92	The back sides of the two left and right rectangular tubes are bent	The rear of the frame	15.75
2	140	The rectangular tube on the right is bent and twisted, the back side of the rectangular tube on the left is bent	The middle of the rectangular tube on the right	13.89
3	268	Right rectangular tube is bent	The middle of the rectangular tube on the right	15.73
4	281	The rectangular tube on the right is bent, and the rear half of the rectangular tube on the left is bent	The rear end of the rectangular tube on the left	12.16
5	443	The front and rear parts of the left rectangular tube are bent separately	The rear of the frame	16.09
6	456	The front and rear parts of the rectangular tubes on the left and right sides are bent respectively	The front end of the rectangular tube on the left	12.15

## 8. Harmonic response analysis of the frame

The modal analysis of the frame indicates that the vibration frequency range for the first six modes is between 92 Hz and 456 Hz. Since the higher frequency end contributes less to structural resonance, there is a significant possibility of severe vibration within this range. In the harmonic response analysis, the frequency segment for analysis is established from 90 Hz to 500 Hz, based on the findings of the modal analysis. A total of 200 steps are calculated within this frequency range.

**Fig 18:**
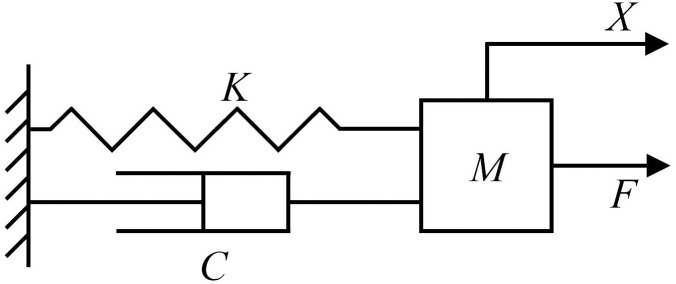
Equivalent effect model.

Harmonic response analysis pertains to the load of a structure, primarily used to ascertain the steady-state response of a linear structure subjected to a simplified load. According to the principles of structural dynamics, the rack can be represented as a continuous physical structure with a mass denoted as *M*. Its system rigidity is represented by *K*, and the damping is indicated by *C*. When an excitation load *F* is applied in the *X*-coordinate direction, the equivalent model can be established as illustrated in Fig 18.

In structural discrete rack finite element models, when the frame is subjected to a simple harmonic load F with a frequency of ω, the persistent vibration micro-division equation is expressed in Equation (8). The moving response of each node can then be determined using Equation (9).


$$\left[M\mathrm{\backslash rightleft}\{\ddot{x}\right\}+\left[C\right]\left\{\dot{x}\right\}+\left[K\right]\left\{x\right\}=\left\{F\left(t\right)\right\}\sin\left(\omega t\right)$$ (8)

{x}={X}sin(ωt+φ) (9)

In the formula, [*m*] denotes the quality matrix, [*C*] represents the damping matrix, [*K*] indicates the stiffness matrix, and {*x*} signifies the displacement vector. The term m; {*F*(*t*)} corresponds to the exciting force (N), while {*X*} denotes the amplitude of the displacement (m). Additionally, *φ* represents the phase angle of the lag in the incentive load, and *t* denotes time (s).

Based on the formula (8) and formula (9), the displacement value of the harmonious response of each node can be obtained (formula (10)).


$$\left\{X\right\}=\frac{\left\{F(t)\text{ sin}(\omega t)\right\}}{\left([K]-[M]\omega^{2}\right)\text{ sin}(\omega t+\varphi )+[C\omega \text{cos }(\omega t+\varphi )}$$ (10)

**Fig 19:**
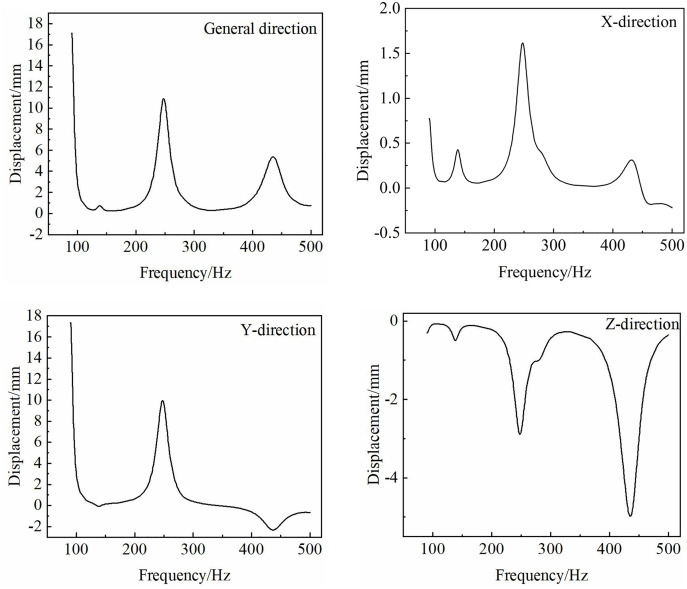
Frequency-displacement curve.

According to the stress on the frame, the loads at the three connections of the tractor are defined as *Z* = 500sin(*ωt*). Utilizing ABAQUS software for a harmonic response analysis, the node with significant displacement changes (tail end) was selected, and a curve chart depicting the frequency variations at the node is generated (Fig 19).

The frequency-displacement curve of the node in the total direction is presented in Fig 19. The figure indicates that the peak of the curve aligns closely with the results obtained from the modal analysis. Notably, the maximum displacements corresponding to frequencies of 92 Hz and 268 Hz are 16.5 mm and 11.1 mm, respectively. Additionally, there are four prominent displacement peaks observed in the x-axis direction, measuring 0.7 mm, 0.4 mm, 1.6 mm, and 0.3 mm. In the y-axis direction, two significant displacement peaks are noted, with values of 16.5 mm and 10 mm. Furthermore, the z-axis direction exhibits four notable displacement peaks, recorded at 0.2 mm, 0.3 mm, 2.9 mm, and 5.1 mm. It is evident that when the frame is subjected to excitation within the frequency range of 90 Hz to 500 Hz, the vibration displacement of the frame remains relatively stable, suggesting that it does not adversely affect the loosening performance of the equipment.

## 9. Conclusion

This article uses numerical simulation to analyze the performance of the plow and the frame respectively. The specific conclusions are as follows:

(1) Based on the discrete element method, the effects of forward speed, lugs angle, and the radius of convex structures on the working resistance of the trencher and the degree of soil disturbance was analyzed. Single-factor and multi-factor experiments were conducted to investigate the influence of three factors: the radius of the mouldboard plough surface convex structure, forward speed, and lugs angle. The responses measured were the working resistance of the trencher and the number of soil disturbance particles. Variance analysis revealed that the degree of influence on soil penetration resistance, ranked from largest to smallest, is as follows: forward speed, lugs angle, and mouldboard plough surface convex radius. Specifically, resistance to soil penetration increases with an increase in forward speed, lugs angle, and the radius of the convex structure. The degree of influence on the number of soil disturbance particles is ranked as follows: forward speed, the radius of the convex structure, and lugs angle. The number of soil disturbance particles decreases with an increase in forward speed, increases with a larger radius of the convex structure, and slightly decreases with an increase in the lugs angle. By establishing a regression model, the optimal parameter combination for the shared mouldboard plough was determined to be a forward speed of 0.8 m/s, a lugs angle of 45°, and a convex structure radius of 9 mm.
(2) Based on finite element analysis, both static and modal analyses were conducted on the frame of the plow device. The results indicated that, during stable operation of the mouldboard plough, the maximum stress occurs at the hinge joint between the frame and the tractor. The maximum stress value recorded is 24.7 MPa, with a corresponding maximum deformation of 0.02 mm, demonstrating that the designed frame satisfies the static requirements. Furthermore, the first-order natural frequency of the frame is 92 Hz, which is significantly higher than the external excitation frequency, thereby preventing the occurrence of resonance.
(3) In the harmonic response analysis of the frame, the excitation frequency ranges from 90 Hz to 500 Hz, with a maximum displacement recorded at 16.5 mm. This indicates that the frame remains relatively stable during the equipment’s working process.
